# Two Bacterial Genera, Sodalis and Rickettsia, Associated with the Seal Louse Proechinophthirus fluctus (Phthiraptera: Anoplura)

**DOI:** 10.1128/AEM.00282-16

**Published:** 2016-05-16

**Authors:** Bret M. Boyd, Julie M. Allen, Ryuichi Koga, Takema Fukatsu, Andrew D. Sweet, Kevin P. Johnson, David L. Reed

**Affiliations:** a Illinois Natural History Survey, University of Illinois at Urbana-Champaign, Champaign, Illinois, USA; b Florida Museum of Natural History, University of Florida, Gainesville, Florida, USA; c Genetics and Genomics Graduate Program, University of Florida, Gainesville, Florida, USA; d Bioproduction Research Institute, National Institute of Advanced Industrial Science and Technology, Tsukuba, Ibaraki, Japan; University of Wisconsin—Madison

## Abstract

Roughly 10% to 15% of insect species host heritable symbiotic bacteria known as endosymbionts. The lice parasitizing mammals rely on endosymbionts to provide essential vitamins absent in their blood meals. Here, we describe two bacterial associates from a louse, Proechinophthirus fluctus, which is an obligate ectoparasite of a marine mammal. One of these is a heritable endosymbiont that is not closely related to endosymbionts of other mammalian lice. Rather, it is more closely related to endosymbionts of the genus Sodalis associated with spittlebugs and feather-chewing bird lice. Localization and vertical transmission of this endosymbiont are also more similar to those of bird lice than to those of other mammalian lice. The endosymbiont genome appears to be degrading in symbiosis; however, it is considerably larger than the genomes of other mammalian louse endosymbionts. These patterns suggest the possibility that this Sodalis endosymbiont might be recently acquired, replacing a now-extinct, ancient endosymbiont. From the same lice, we also identified an abundant bacterium belonging to the genus Rickettsia that is closely related to Rickettsia ricketsii, a human pathogen vectored by ticks. No obvious masses of the Rickettsia bacterium were observed in louse tissues, nor did we find any evidence of vertical transmission, so the nature of its association remains unclear.

**IMPORTANCE** Many insects are host to heritable symbiotic bacteria. These heritable bacteria have been identified from numerous species of parasitic lice. It appears that novel symbioses have formed between lice and bacteria many times, with new bacterial symbionts potentially replacing existing ones. However, little was known about the symbionts of lice parasitizing marine mammals. Here, we identified a heritable bacterial symbiont in lice parasitizing northern fur seals. This bacterial symbiont appears to have been recently acquired by the lice. The findings reported here provide insights into how new symbioses form and how this lifestyle is shaping the symbiont genome.

## INTRODUCTION

The sucking lice (Anoplura) are obligate and host-specific ectoparasites of mammals. These lice have specialized piercing-sucking mouthparts for feeding exclusively on mammal blood (1). One of these louse species, the human body louse Pediculus humanus, has been shown to rely on endosymbiotic bacteria for normal development that supply the lice with B vitamins deficient in vertebrate blood (reference 2, as interpreted by Perotti et al. [3]).

Heritable endosymbiotic bacteria (all belonging to the Enterobacteriaceae in the Gammaproteobacteria) are known for many species of mammalian sucking lice (4–17). These bacteria are housed in specialized organs, known as bacteriomes, and are vertically transmitted from mother to offspring (10, 11, 16, 18, 19). However, the location, general characteristics, and tissue type constituting the bacteriomes may differ across louse lineages (4, 10). On the basis of these observations, Buchner (10) suggested that the bacteriomes had originated independently in different louse lineages and were not derived from an ancestral structure. Later, molecular phylogenetics showed that the bacteria inhabiting these bacteriomes are distantly related to each other (14–17; see also reference 20 for a review). These patterns suggest that louse-bacterium endosymbiotic associations have originated multiple times and/or that the endosymbionts have been replaced repeatedly.

Despite the growing knowledge of mammal-louse endosymbioses, little is known about the endosymbionts of sucking lice parasitizing marine mammals. In total, four genera of sucking lice are known to parasitize walruses and seals (21). We obtained specimens of the seal louse, Proechinophthirus fluctus, from the northern fur seal, Callorhinus ursinus, a marine mammal found in the Bering Sea, Pacific Ocean, and Sea of Japan (22). These lice are specialized for parasitizing marine mammals and, unlike lice on land animals, may have limited opportunities to feed (23). Upon initial inspection, these lice exhibited no obvious bacteriomes like those found, for example, in human lice.

The goals of this study were to determine whether these fur seal lice harbor endosymbionts and, if so, to identify these bacterial associates. In order to characterize potential bacterial endosymbionts of the northern fur seal louse, P. fluctus, we adopted a variety of techniques, including molecular phylogenetics using multiple markers, genome sequencing, *in situ* hybridization targeting bacterial 16S rRNA, and assessment of pathogenicity, B-vitamin synthesis, and motility as predicted by genome annotation data.

## MATERIALS AND METHODS

### Sample collection.

Proechinophthirus fluctus Osborne (Phthiraptera: Anoplura) fur seal lice were collected from Callorhinus ursinus Linnaeus (Otariidae) fur seals in St. Paul Island rookery, Pribilof Islands, Alaska, USA. Lice were preserved in 95% ethanol and stored at −80°C.

### DNA extraction and sequencing.

Genomic DNA was extracted and isolated from whole lice using a phenol-chloroform method (24). DNA extracts from four lice were pooled, and total genomic DNA was used in library construction. The library was constructed using the Illumina TruSeq sample preparation kit with a targeted insert size of 300 to 400 bp. DNA fragments were sequenced on one-half lane of the Illumina HiSeq 2000 platform using the TruSeq SBS sequencing kit, yielding 100-bp paired-end reads. Library preparation and sequencing were done at the W. M. Keck Center, University of Illinois at Urbana-Champaign.

### Genome assembly.

We largely followed the symbiont genome assembly methods described by Boyd et al. (24). This included removing suspect base calls in the Illumina paired-end read library by quality trimming. To do this, we trimmed the first five bases from the 5′ end of each read and seven bases from the 3′ end to remove read positions that had elevated AT content. The reads were then soft trimmed from the 3′ end to remove base calls with a phred score of less than 28 using a sliding window of 1 nucleotide. Reads that had fewer than 75 bp after quality trimming were removed from the library along with their mates. Remaining reads were assembled *de novo* into contigs using ABySS genome assembler (k = 64, paired-end) (25). To identify contigs representing the endosymbiont genome, all contigs were compared to a library of bacterial genomes, including gamma- and alphaproteobacterial endosymbionts and Gram-positive bacterial species, using blastn (including “Candidatus Riesia pediculicola” strain USDA gi295698239 and gi292493920; Sodalis glossinidius strain morsitans gi85057978, gi85060411, gi85060466, and gi85060490; Wigglesworthia glossinidia gi32490749, gi19225058, and gi19225058; Photorhabdus luminescens subsp. laumondii gi37524032; Yersinia pestis gi31795333; Bacillus subtilis subsp. subtilis gi223666304; Buchnera aphidicola strain APS gi15616630, gi10957103, and gi10957099; “Candidatus Blochmannia floridanus” gi33519483; and Rickettsia conorii strain Malish 7 gi15891923) (26). Contigs showing significant similarity (determined by an expected value approaching zero) to these bacterial genomes were considered to be part of an endosymbiont genome that had assembled into contigs independently of the louse genome.

Our initial alignment of contigs to bacterial genomes suggested that two species of bacteria were present in the lice. One was an alphaproteobacterium belonging to the genus Rickettsia, and the other was a gammaproteobacterium that shared high sequence similarity to Sodalis glossinidius. Before reconstruction of these bacterial genomes, we needed to separate sequence data belonging to each bacterial genome. We isolated all reads that were incorporated into the assembly of each bacterial contig identified in the *de novo* assembly described above. This was done by aligning reads to the contigs using bowtie2 v.beta2.6 alignment options –end-to-end and –sensitive and printing aligned reads to the file using the –al-conc option (27). This subset of the original reads was assembled independently of the louse sequence data using ABySS. The resulting contigs were then compared to representative genomes of Rickettsia (R. conorii strain Malish 7 gi:15891923) and Sodalis (S. glossinidius strain morsitans gi:85057978) using blastn. Contigs were assigned either to Rickettsia or Sodalis based on blast scores (i.e., those contigs with a significantly better alignment score to the R. conorii genome and lower E value were considered to belong to the Rickettsia genome and vice versa). Reads that went into the construction of these contigs were then again identified using read mapping and assigned to either the Rickettsia genome or the Sodalis genome. From these reads, both genomes were assembled independently using ABySS. Draft genome assemblies were annotated using the rapid annotation subsystem pipeline (RAST) available at rast.nmpdr.org (submission date for Sodalis genome, 21 November 2012; submission date for Rickettsia genome, 27 November 2012) (28, 29). The resulting annotated genomes were downloaded from RAST as GenBank flat files and as general feature format files. The general feature files along with fasta files of the contigs from the Sodalis endosymbiont were loaded into SyMAP for a whole-genome comparison with other Sodalis genomes (30, 31).

To evaluate sequencing depth and genome variation in the Sodalis genome, we again aligned the reads to the assembled genomic contigs using bowtie2 (same alignment settings as described above). The results were output to a sequence alignment map (SAM) file and converted to its binary equivalent (BAM file) using the SAMtools view function (32, 33). The BAM file was manually viewed in Geneious (Biomaters). Awk and Geneious were used to determine the genomic mean and standard deviation of sequencing coverage as well as to identify significantly high and low coverage regions. SAMtools (mpileup) and bcftools (view) were used to generate a VCF file, and this file was filtered to include single nucleotide polymorphism (SNP) calls only.

Subsequent investigation identified that the Rickettsia bacterium was closely related to Rickettsia peacockii. R. peacockii carries a 26,406-bp plasmid that was not in our assembly (34). To determine if the plasmid was present in our read library but failed to assemble, we aligned reads to paralogs of the *dnaA* gene found in the primary chromosome and the plasmid in R. peacockii. The sequences of these two genes are considerably different; if the plasmid was present, we would expect to see variation in sequence reads containing the gene. Therefore, reads would align to each paralog using Bowtie2 vbeta2.6 using options –end-to-end and –sensitive, demonstrating that two copies of the gene were present in the library. If the sequence data aligned to only one gene, we assumed that only one copy was present. Sodalis species may also harbor plasmids, and we used blastn (v. 2.2.28) to search our genome assembly against known Sodalis plasmids (gi85060490, gi85060466, gi85060411, gi749309516).

### Identifying Rickettsia genes involved in pathogenicity.

Felsheim et al. (34) identified genes whose products may be involved in host cell invasion and virulence in the spotted fever group of Rickettsia species. On the basis of their report, we obtained sequences of these genes from the Rickettsia rickettsii Sheila Smith (SS) genome using the gene identifiers reported by Felsheim et al. (34). We used a tblastx search to find potential orthologs shared between R. rickettsii SS and genes annotated by RAST in our Rickettsia bacterium. BLAST results were filtered for bidirectional best hits to the virulence genes in R. rickettsii identifiers reported by Felsheim et al. (34). These genes were aligned to their respective orthologs in R. rickettsii SS using Muscle, and alignments were viewed in Geneious to visually identify differences in gene sequences between the species (35).

### Sodalis insertion sequence search.

To find known Sodalis insertion sequences (IS) in this endosymbiont genome, we searched for known IS identified in two other Sodalis genomes. We downloaded IS from NCBI for Sodalis endosymbiont strain PSPU IS1 (gi612149276 locus tag AB849119) and “Candidatus Sodalis pierantonius” strain SOPE ISsope1 to -4 (gi218090034 locus tags AM921789.1, AM921790.1, AM921791.1, and AM921792.1, respectively). The Sodalis endosymbiont contigs were used to build an NCBI blast database in Geneious. We then searched for the IS in the Sodalis endosymbiont contigs using tblastn. To search for novel IS, we compared the genome to itself using SynMap implemented in CoGe (https://genomeevolution.org) (36, 37). The genome was loaded on both the *x* and *y* axes, and the syntenic path assembly option was selected. The results were visualized in CoGe, searching for sequences with hits in multiple locations across the contigs.

To determine if the IS were present in our read library but failed to assemble as part of the Sodalis genome, we used aTRAM (38). This software uses a reference sequence to search for similar reads using blast and conducts a *de novo* assembly of those reads. We used the Illumina HiSeq read library as the short read sequences and the five IS sequences described above as the references and chose ABySS as the *de novo* assembler.

### Predicted B-vitamin synthesis by Sodalis.

Human louse endosymbionts are believed to provide B vitamins to their louse hosts (2, 3). To determine if the Sodalis genome has genes encoding proteins involved in B-vitamin synthesis, we built two complete metabolic models for this organism. A metabolic model was first constructed using the RAST pipeline (29). RAST uses an initial automated annotation to identify features within a new genome, establishes a phylogenetic context for the organism, and finally compares the annotation to a set of manually curated genes from closely related organisms for annotation validation and correction (29). These data were compiled into an initial metabolic reconstruction for the organism. We viewed cofactor and vitamin subsystems within this complete model using the SEED Viewer (28, 39). We then used SEED tools to compare the genome of the new endosymbiont to the S. glossinidius genome (the only other Sodalis genome available for comparison at the time). Genes present in the S. glossinidius genome associated with B-vitamin synthesis but not found in the initial metabolic model for our new endosymbiont were manually searched for by using blast tools within SEED. Next, we built a second metabolic model for this endosymbiont using the KAAS server (40). To do this, we exported all amino acid sequences from the RAST annotation server to a fasta file. These data served as the starting data for creating the new KAAS model. This was necessary, as KAAS does not include tools for gene discovery. Instead of using phylogenetic estimations (like those done by RAST) for comparison and annotation of genes, KAAS uses a default set of genomes for gene comparison (40). We added genome data from B. aphidicola, “*Ca*. Riesia pediculicola,” S. glossinidius, and W. glossinidia to the default genome list of compared genomes. We then compared resulting metabolic models for pantothenate, thiamine, folate, riboflavin, pyridoxine, and biotin synthesis to the corresponding models generated by the RAST subsystem. Results from the two methods were viewed by painting present functions onto KEGG pathway maps.

Upon review of the results, we identified significant differences in the metabolic prediction for this endosymbiont from that of the newly published and closely related Sodalis praecaptivus genome. Gene sequences whose products were involved in B-vitamin biosynthesis in S. praecaptivus were downloaded from KEGG using WebDBGET (41–46). This included predicted genes *ilvG*, *ilvM*, *ilvH*, *ilvI*, *ilvE*, *ilvD*, *ilvC*, *panE*, *panB*, *panC*, *panD*, *coaA*, *dfp*, *coaD*, *coaE*, *folC*, *pdxA*, *pdxY*, *fabB*, *fabF*, *fabG*, *fabZ*, *fabI*, *bioH*, *bioF*, *bioA*, *ynfK*, *bioB*, *thiC*, *thiE*, *thiF*, *thiS*, *thiG*, and *thiH*. We then used blastn and tblastx searches to identify candidate genes in the fur seal louse endosymbiont genome. The results were then used to manually identify and develop annotations for these genes. The gene *coaA* was expected to be present but could not be identified in the genome assembly. Therefore, an independent aTRAM assembly (38) was conducted for this gene to determine if it was absent or missing due to an assembly error.

### Sodalis motility.

*In situ* hybridization of Sodalis cells in fur seal lice, described below, identified extracellular cells with large comet-like tails. Two structures could result in this phenotype, flagella or actin filaments. S. praecaptivus genes encoding flagellar structural and regulatory proteins were downloaded from KEGG using WebDBGET. These genes were searched for in the fur seal Sodalis genome using both blastn and tblastx. BLAST results were edited manually and used to annotate S. praecaptivus flagellum-associated gene products present in the fur seal Sodalis endosymbiont (see Table S1 in the supplemental material). Those genes that were found to span regions of a contig estimated by the genome assembly software using n's or spanning two contigs were assumed to be intact. The other structure that could appear as comet tails is actin filament and is known from more distantly related bacteria, such as Listeria (47). We used tblastx to search for a gene, *actA* (from Listeria), involved in actin assembly, in the S. praecaptivus and seal louse–associated Sodalis genomes.

### Phylogenetic reconstruction.

To determine the phylogenetic relationship of the Sodalis endosymbiont within the gammaproteobacterial family Enterobacteriaceae, we built a maximum likelihood tree based on three protein coding genes (*groEL*, *dnaJ*, and *ftsZ*) from representative Enterobacteriaceae (including Sodalis species described in Table 1) and two outgroup taxa from Pasteurellaceae. We selected *dnaJ* and *ftsZ*, because they are single orthologs present in >80% of Enterobacteriaceae genomes on orthoDB v8 (48) and could be retrieved from sequenced genomes. The gene *groEL* was chosen because this gene had been sequenced for additional Sodalis species for which there were no genome data available. Gene sequences for all three genes were retrieved from EnsembleBacteria release 26 or NCBI for taxa that had publically available genomes. Additional *groEL* sequences were obtained from nine Sodalis species (Table 1) and a chewing louse endosymbiont not closely related to Sodalis (clade C endosymbiont, described in reference 49). The gene sequences were aligned using Muscle, implemented in Geneious. The sequences were concatenated by taxon, and missing genes were coded as gaps. PartitionFinder (v1.1.1) was used to find the best model of sequence evolution for each gene from all possible models using the AICc correction (50). A maximum likelihood tree was constructed using RAxML (VI-HPC) using a GTR+Γ+I model of sequence evolution for each partition (51). Support for the most likely tree was determined by percentage of 1,000 rapid bootstrap replicates implemented in RAxML. The tree was rooted to Pasteurella and Haemophilus and viewed in FigTree (http://tree.bio.ed.ac.uk/software/figtree/). Due to much higher taxon sampling within Sodalis with the *groEL* gene, a second maximum likelihood tree was built using only the aligned *groEL* sequences with RAxML. We used a GTR+Γ+I model and determined support by percentage of 1,000 bootstrap replicates. The tree was rooted to Pasteurella and Haemophilus and viewed in FigTree.

**TABLE 1:** Sodalis species used in phylogenetic analysis

Sodalis taxonomic classification	Strain	Host classification		Data availability			
		Host	Host common name	Genome	*groEL*	*dnaJ*	*ftsZ*
Sodalis glossinidius	Morsitans	Glossinia morsitans	Tsetse fly	Yes	Yes	Yes	Yes
“*Ca*. Sodalis melophagi”		Melophagus ovinus	Sheep ked fly	No	Yes	No	No
Sodalis endosymbiont		Proechinophthirus fluctus	Northern fur seal louse	Yes	Yes	Yes	Yes
Sodalis endosymbiont		Columbicola exilicornis	Cuckoo-dove louse	No	Yes	No	No
Sodalis endosymbiont		Columbicola columbae	Rock dove louse	No	Yes	No	No
Sodalis endosymbiont		Columbicola macrourae	Mourning dove louse	No	Yes	No	No
“*Ca*. Sodalis pierantonius”	SOPE	Sitophilus oryzae	Rice weevil	Yes	Yes	Yes	Yes
Sodalis endosymbiont	PSPU	Philaenus spumarius	Meadow froghopper	Yes	Yes	Yes	Yes
Sodalis endosymbiont		Aphrophora quadrinotata	Four-spotted spittlebug	No	Yes	No	No
Sodalis endosymbiont		Philaenarcys bilineata	Prairie spittlebug	No	Yes	No	No
Sodalis endosymbiont		Mesoptyelus fascialis	Spittlebug	No	Yes	No	No
Sodalis endosymbiont	NRF1	Antestiopsis thunbergii	Stinkbug	No	Yes	No	No
Sodalis endosymbiont		Cantao ocellatus	Shield bug	No	Yes	No	No
Sodalis praecaptivus	HS1	NA^*a*^: free-living/pathogenic	NA^*a*^	Yes	Yes	Yes	Yes

a NA, not applicable.

To determine the phylogenetic placement of the Rickettsia bacterium associated with the fur seal louse, we used three protein coding genes, *atpA*, *coxA*, and *gltA*, to build a phylogenetic tree of Rickettsia species. These genes were identified by Weinert et al. (52) as useful phylogenetic markers for Rickettsia species and were available in sequenced genomes. The three protein coding genes were identified in our draft genome sequence and in other Rickettsia and *Orientia* genomes using tblastx through the CoGe platform (accessed through http://genomevolution.org) (36, 37). Gene sequences were aligned using Muscle, implemented in Geneious. Aligned gene sequences were then concatenated to form a single matrix. As with the Enterobacteriaceae analysis, a maximum likelihood tree was constructed in RAxML (VI-HPC) from this matrix, under a GTR+Γ model of sequence evolution. Support for the tree was assessed from percentage of 1,000 rapid bootstrap replicates implemented in RAxML. The tree was rooted to *Orientia* and viewed in FigTree.

### *In situ* identification of endosymbionts.

The 16S rRNA gene was isolated and resequenced for both bacterial species from three additional lice using global primers 27F, 1525R, and 1329R (53). PCR was done using Stratagene high-fidelity master mix. PCR products were cloned using an Invitrogen cloning kit, and 96 colonies were sequenced using Sanger sequencing. Alignment of Illumina reads to the resulting Sanger sequence verified that they were identical. These Sanger-derived 16S rRNA genes were used to construct probes to identify both endosymbionts using *in situ* florescent hybridization of tissue sections.

Acetone-preserved lice (54) were rehydrated in Dulbecco's phosphate-buffered saline (PBS; Sigma), and their heads, legs, and posterior ends of the abdomens were removed to facilitate infiltration of reagents. Then, the tissues were fixed in PBS containing 4% formaldehyde (Wako Chemicals) at 4°C overnight. After several washes with PBS, the fixed tissues were dehydrated with several washes with acetone, followed by a 1-h incubation. The dehydrated tissues were embedded in a resin of the Technovit 8100 embedding kit (Kulzer). Thin sections of 2-μm thickness were cut by using an RM2165 (Leica)rotary microtome. The sections were fixed on MAS-coated glass slides (Matsunami) and kept at −20°C until labeling.

The fluorescence-labeled probe Al555-Sod181R (5′-CAC TTT GGT CTT GCG ACA T-3′) specific to the Sodalis endosymbiont was designed manually with assistance from the Silva database (55) and Probebase (56) and was labeled with Alexa Fluor 555 (Al555) at its 5′ end. Hybridization of the tissue sections was conducted using a buffer containing 10 nM Al555-Sod181R and also 10 nM Alexa Fluor 647-labeled universal probe EUB917 (5′-GGG YCC CCG YCA ATT C-3′). This buffer was applied to the tissue sections on a slide glass and incubated at 25°C overnight in a humidified chamber. Then, the sections were subjected to three successive washes with PBS containing 0.1% Tween 20 (PBST) for 10 min each and were mounted in ProLong gold antifade reagent (Life Technologies). The mounted sections were observed and photographed on an Axiophot2 (Zeiss) microscope system. Digital images were merged, and the contrast was adjusted using Photoshop CS5.1 (Adobe).

### Nucleotide sequence accession numbers.

Genome assemblies referenced in the manuscript have been deposited in DDBJ/ENA/GenBank (www.ncbi.nlm.nih.gov), with Sodalis sp. strain SPI-1 genome contigs deposited under accession no. LECR00000000 (version LECR01000000) and Rickettsia sp. strain SPI-2 genome contigs deposited under accession no. LECS00000000 (version LECS01000000).

## RESULTS

### Phylogenetic placement.

**FIG 1:**
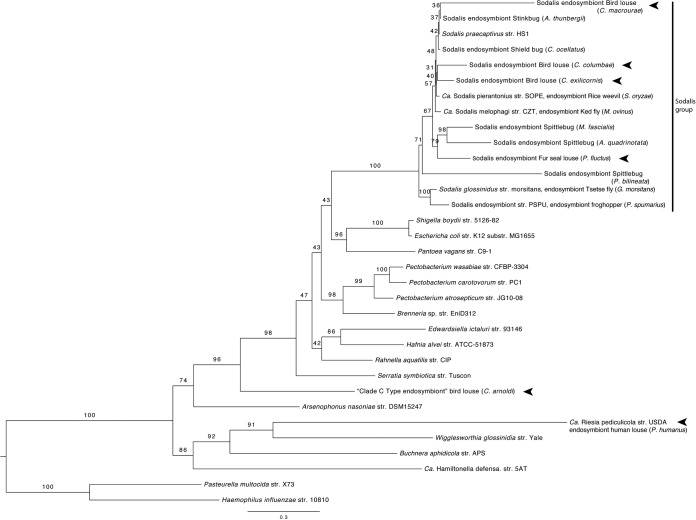
Fur seal louse endosymbiont supported as a member of the Sodalis clade in the most likely tree based on maximum likelihood analysis of combined *groEL*, *dnaJ*, and *ftsZ* sequences from Sodalis and allied endosymbionts, representative Enterbacteriaceae, and outgroup taxa from Pasteurellaceae. Numbers at nodes are percentages of 1,000 bootstrap replicates. A vertical bar delineates the Sodalis group of bacteria, and arrowheads indicate louse endosymbionts. The clade C type endosymbiont from bird lice (Columbicola arnoldi) represents an Enterobacteriaceae endosymbiont distantly related to Sodalis described by Smith et al. (49). Other bird louse endosymbionts were described by Smith et al. (49) as belonging to clade A from Columbicola, a Sodalis clade.

Phylogenetic reconstruction of representative Enterobacteriaceae including the new fur seal louse endosymbiont found that this new endosymbiont was a member of the Sodalis and allied endosymbiont clade. This was true for both the concatenated three-gene matrix (*groEL*, *dnaJ*, and *ftsZ*) and the *groEL* sequences analyzed alone (Table 1 and Fig. 1; see Fig. S1 in the supplemental material). Support for this clade was high, with 100% of 1,000 bootstraps recovering this clade. The relationships among species within the Sodalis clade were largely unresolved, and bootstrap support for branches within the clade was moderate to low (most nodes were supported with <75% of bootstrap replicates). The other mammal louse endosymbiont included in this analysis, the human louse endosymbiont (*Ca*. Riesia pediculicola), was found to be distantly related to Sodalis species. It was more closely related to W. glossinidia and B. aphidicola in the tree built from the three-gene matrix and to A. nasoniae when a tree was constructed using *groEL* alone.

**FIG 2:**
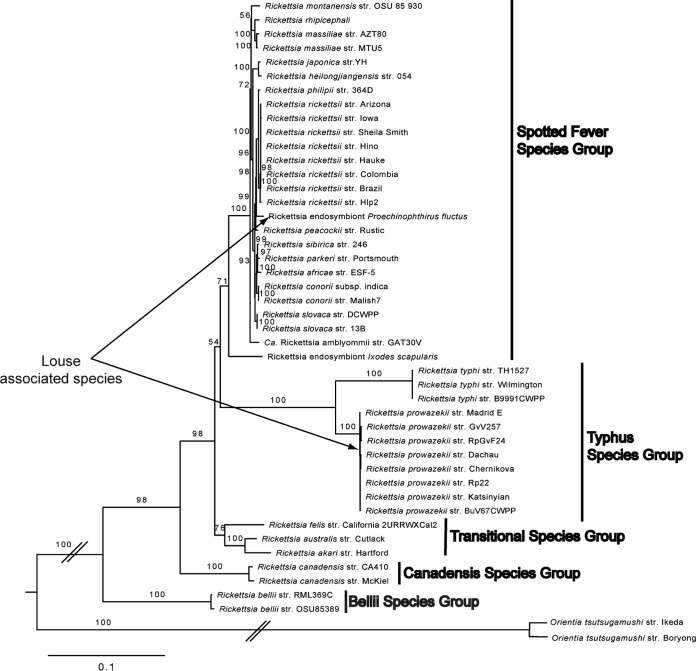
The fur seal louse-associated Rickettsia bacterium supported a member of the spotted fever species group in the most likely tree based on maximum likelihood analysis of *atpA*, *coxA*, and *gltA*, showing relationships of Rickettsia and *Orientia* (Rickettsiaceae). Numbers at nodes are percentages of 1,000 bootstrap replicates. Vertical bars delineate Rickettsia species groups. Louse-associated Rickettsia species are indicated by arrows. The slashes indicate branch lengths that were shortened for publication.

Phylogenetic reconstruction of Rickettsia species based on three protein coding genes supported the fur seal louse-associated Rickettsia bacterium as a member of the spotted fever species group. Specifically, it was placed in a clade that includes Rickettsia rickettsii, Rickettsia philipii, and Rickettsia peacockii (Fig. 2). It was not supported as belonging to the typhus clade species that has been previously found in human head lice. When estimating phylogenetic relationships from protein coding genes, we found strong support (>90% of 1,000 bootstrap replicates) for this relationship.

### Genome assemblies.

The draft genome assembly of the Sodalis endosymbiont totaled 2,179,576 bp, and 50% of bases were G or C. The assembly resulted in 99 scaffolds, with the largest being 171,838 bases long. The mean genome-wide read coverage was 29×, with a standard deviation (SD) of 16. A detailed review of the longest contig revealed regions of high and low coverage. Mean read coverage for this contig was slightly higher than the genomic average (mean = 32×, SD = 15), but 4.2% of the contig bases had very high coverage and 1.6% of the bases had very low coverage (more than 2 SD). The length of high- and low-coverage regions was smaller than the read and insert sizes (mean size = 50 bp, range = 1,339 bp; read length = 75,100 bp; insert target range for paired reads = 300 to 400); therefore, these are regions that could be estimated from read associations during genome assembly. This suggests that genomic build may be fragmented due to inconsistencies in genome sequencing depth and that the contigs are likely separated by assembly gaps greater than 400 bp. Despite potential gaps in the genome assembly, the size of our genome build is within the range of other sequenced Sodalis genomes (Sodalis sp. strain PSPU at 1.4 Mbp, 54% GC; S. glossinidius strain moristans at 4.5 Mbp, 55% GC; “*Candidatus* Sodalis pierantonius” strain SOPE at 4.5 Mbp, 56% GC; and the free-living S. praecaptivus strain HS1 at 4.7 Mbp, 57% GC) (57–60). This is also considerably larger than previously sequenced AT-rich genomes of human and chimpanzee louse endosymbionts (“Candidatus Riesia” species: size = 0.577 Mbp and 0.582 Mbp, respectively; % GC = 37% and 35%, respectively) (24, 61).

Another issue that may cause the genome assembly to be fragmented is the presence of genomic variation in the bacteria sampled for sequencing. In order to obtain enough DNA for genome sequencing, we had to pool extracts from four lice collected from the same mammalian host. Upon review of the data, we identified the presence of SNPs in the Sodalis genome data. In total, we identified 2,034 candidate SNPs spread out through the contigs. However, these did not appear to have affected the quality of the assembly, as many SNPs were found within accurately assembled genome regions and were called with either one of the alternative alleles or by an ambiguous base by the assembly software.

**FIG 3:**
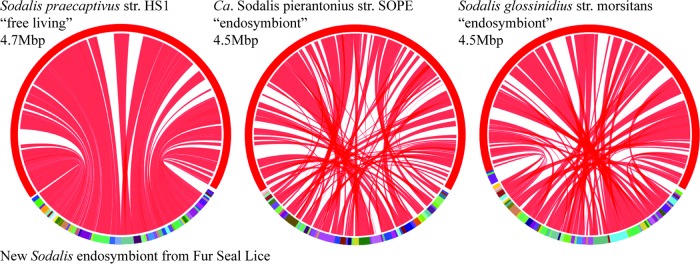
Comparison of Sodalis genomes highlighting syntenic regions. The lower half of the circle represents the fur seal louse endosymbiont contigs, and the upper half represents other Sodalis genomes. Shading between genomes indicates shared syntenic blocks based on coding regions. Colors delineate different contigs or chromosomes, and color choice is arbitrary. Seal louse endosymbiont contigs are ordered by their synteny with the genome to which they are compared, but the true order and orientation of the louse contigs are not known because of the fragmented nature of the genome assembly. Images were generated using SyMAP (30, 31).

Nearly all of the contigs from the Sodalis endosymbiont genome showed genomic synteny with the Sodalis praecaptivus strain HS1 genome (Fig. 3). Genomic syntenies between the fur seal louse Sodalis endosymbiont and the “*Candidatus* Sodalis pierantonius” strain SOPE and S. glossinidius strain morsitans endosymbionts were slightly less complete (Fig. 3). None of the contigs showed a similarity to the plasmids known from other Sodalis species. We failed to detect insertion sequences previously known from “*Candidatus* Sodalis pierantonius” strain SOPE and S. glossinidius strain morsitans either in our assembly or in the original sequencing library.

**FIG 4:**
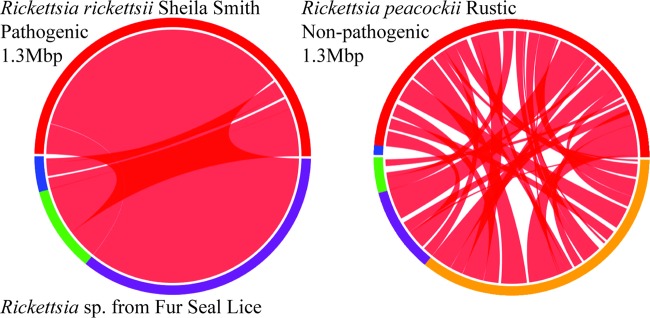
Comparison of the fur seal louse-associated Rickettsia genome with the genomes of pathogenic R. rickettsii Sheila Smith and the endosymbiont R. peacockii Rustic. The lower halves of the circles represent the fur seal louse-associated Rickettsia genome, and the upper half represents previously described Rickettsia genomes. Shading between genomes indicates shared syntenic blocks, as determined by coding regions. Colors delineate different contigs or chromosomes, and color choice is arbitrary. Images were generated using SyMAP (30, 31).

The draft genome sequence of our Rickettsia bacterium totaled 1,251,943 bp and was 32% GC. The genome was assembled into 3 larger scaffolds that contained most of the genome and 10 smaller scaffolds. We found no evidence of the pRPR plasmid known from the closely related species R. peacockii. Alignment of our scaffolds showed that gene order was largely conserved between our new louse-associated Rickettsia bacterium and R. rickettsii, with the exception of two inversions (Fig. 4). One was a large inversion that occurred in the common ancestor of our new Rickettsia bacterium, R. rickettsii, and their sister species, R. parkerii, with a subsequent inversion within the original inversion in R. rickettsii. The second inversion appeared unique to the new seal louse Rickettsia bacterium.

### Endosymbiont characteristics.

**FIG 5:**
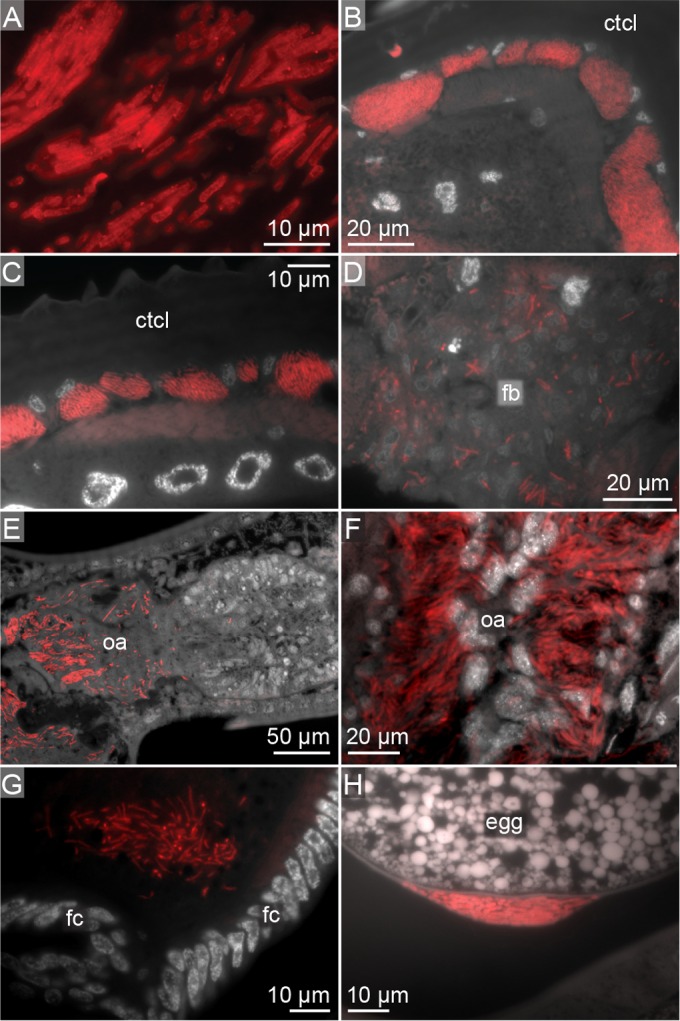
Storage and transmission of Sodalis endosymbionts. Red indicates *in situ* hybridization of 16S rRNA in cross sections of lice. (A) Sodalis endosymbiont cells; (B and C) intracellular masses of Sodalis cells in bacteriocytes along the abdominal wall; (D) diffuse Sodalis cells in the fat body; (E and F) Sodalis cells in lateral oviducts; (G and H) masses of Sodalis cells in posterior pole of oocytes. ctcl, cuticle; fb, fat body; fc, follicle; oa, ovary.

**FIG 6:**
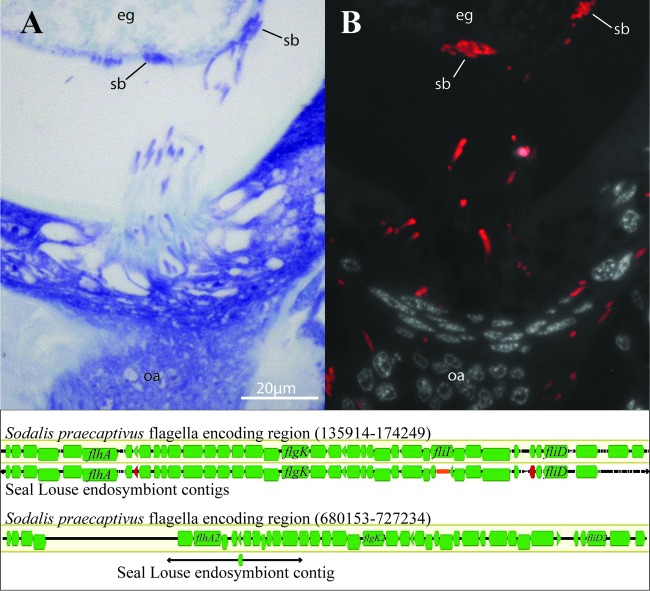
Locomotion of Sodalis endosymbionts during infection of oocytes and comparison of predicted flagellum-encoding genomic regions in Sodalis species. (Top) Cross section of adult louse showing Sodalis cells with comet-like tails migrating from the lateral oviduct to the posterior pole of the oocyte with toluidine staining (A) and the same cross section with binding of the Sodalis 16S rRNA probe (B). (Bottom) Comparison of flagellum-encoding genomic regions between S. praecaptivus and the fur seal louse endosymbiont with genes involved in the production of flagella annotated in green (only representative genes identified by name) and candidate pseudogenes annotated in red. An orange bar represents the estimated gap between contigs. eg, egg; sb, symbiont ball; oa, ovary.

The Sodalis endosymbiont cells were rod shaped like S. preacaptivus but, at ∼6 μm in length and ∼1 μm in diameter, were larger than S. preacaptivus cells (Fig. 5A). These endosymbiont cells were densely packed in bacteriocytes found throughout the wall of the abdomen, while lower concentrations of endosymbiont cells were observed in abdominal fatty tissues (Fig. 5B to D). Additional infections were found in the lateral oviducts of adult female lice (Fig. 5E and F). In one of the specimens, Sodalis endosymbiont cells were found to have escaped the tissues of the lateral oviduct and were migrating to the posterior pole of a developing egg. These extracellular cells exhibited comet-like tails (Fig. 6A and B). Large masses of Sodalis endosymbiont cells were found in the posterior pole of eggs (Fig. 5G and H). This is consistent with maternal inheritance of Sodalis endosymbionts, which is the primary method of transmission in other Sodalis species (62–68). After migrating to the oocytes, the Sodalis cells invade and form a large mass at the posterior pole of the developing eggs (Fig. 5G and H). This is similar to chewing louse (Sodalis species) and human louse (“*Ca*. Riesia pediculicola”) endosymbionts that also invade through the posterior pole (11, 12). Perotti et al. (12) found that the pores in the hydropyle provided the route for human louse endosymbiont invasion at the posterior pole. This Sodalis endosymbiont may also be utilizing these same pores for oocyte invasion. We were unable to locate Rickettsia cells using the same techniques, suggesting that they may be more diffusely associated with the louse tissues or enteric.

### Motility by Sodalis.

As described above, the Sodalis endosymbionts exhibit large comet-like tails. Either flagella or actin filaments could result in this observed phenotype. The related free-living S. praecaptivus endosymbionts possess two larger genomic regions that both contain all of the genes needed to form flagella and to regulate their production (58). We identified a region in the fur seal louse Sodalis endosymbiont that was potentially orthologous to one of these regions. In this region, we identified S. praecaptivus genes that encode flagellar structural components and positive regulators of flagellar synthesis, including *flgA*, *flgB*, *flgC*, *flgD*, *flgE*, *flgF*, *flgG*, *flgH*, *flgI*, *flgK*, *flgL*, *flhA*, *flhB*, *flhC*, *flhD*, *fliC1*, *fliC2*, *fliD*, *fliE*, *fliF*, *fliG*, *fliH*, *fliI*, *fliJ*, *fliK*, *fliM*, *fliN*, *fliO*, *fliP*, *fliQ*, *fliR*, *fliS*, *motA*, and *motB* (Fig. 6; see Table S1 in the supplemental material). However, unlike this region in S. praecaptivus, the genes *flgM*, a negative regulator of flagellar synthesis, and *fliT* were absent (although candidate pseudogenes for each were identified). We did find a potential ortholog of the S. praecaptivus gene *flgM2*, the negative regulator gene found in the second S. praecaptivus flagellum-encoding region. None of the other genes in this second region were detected. We failed to identify a candidate ortholog of the *actA* gene, a gene essential to actin filament formation (69), in either S. praecaptivus or the fur seal louse endosymbiont.

### B-vitamin synthesis in Sodalis.

**FIG 7:**
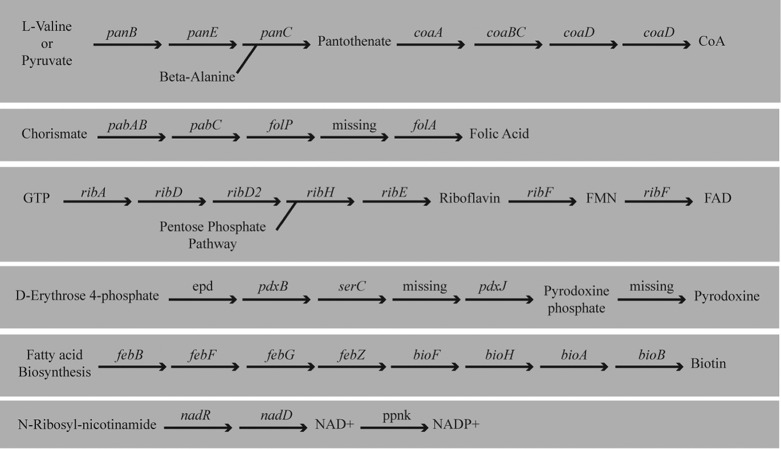
Biosynthetic pathways for B vitamins and cofactors as predicted by the genomic data for the Sodalis endosymbiont of the fur seal louse.

Metabolic reconstruction using both RAST and KAAS models and manual annotation against the S. praecaptivus genome supported the Sodalis endosymbiont as possessing complete pathways for synthesis of pantothenate, folate, nicotinamide, riboflavin, biotin, and pyridoxine (Fig. 7; see Tables S1 and S2 in the supplemental material).

*De novo* biosynthesis of pantothenate (vitamin B_5_; transportable precursor of coenzyme A) from 3-methyl-2-oxobutanoate and β-alanine appeared complete, with the genes *panB*, *panC*, and *panE* being present. Synthesis of 3-methyl-2-oxobutanoate from either pyruvate or l-valine appeared complete; however, the *panD* gene required for synthesis of β-alanine was not predicted. Using blast results of *panB*, *panC*, *panD*, and *panE* genes, we identified a genomic region containing candidate *panB*, *panC*, and *panD* orthologs located at a distance from *panE*, an arrangement similar to that of S. praecaptivus. The *panD* gene was found in blast searches but was not predicted in the RAST model. By aligning the S. praecaptivus panD sequence to this region, we isolated the endosymbiont *panD* ortholog and translated it to find two stop codons within the gene (see data in the supplemental material). This suggests that the endosymbiont has lost the ability to synthesize β-alanine through loss of the *panD*-encoded function. RAST and KAAS predicted that synthesis of coenzyme A from pantothenate was largely intact with *dfp*, *coaD*, and *coaE* present but with *coaA* missing. Subsequent blast searches using the S. praecaptivus *coaA* gene sequence also failed to find this missing gene. However, an independent aTRAM assembly yielded much of this gene sequence (see data in the supplemental material). Thus, it is likely that *coaA* is present but appears to be missing due to assembly error.

Synthesis of folate (vitamin B_9_) from chorismate or 7-8-dihydrofolate appeared to be present, but synthesis of folate from GTP appeared incomplete, with the genes *folB*, *folC*, and *folK* absent. An additional blast search for *folC* failed to identify this gene, but given the completeness of the pathway, it may also be missing due to an assembly error. Elements of the phosphorylation pathway to produce cofactors NAD and NADP from nicotinamide d-ribonucleotide (vitamin B_3_) were present, though limited in comparison to other Sodalis species, with only the genes *nadE*, *nadR*, and *ppnK* present. Synthesis of riboflavin (vitamin B_2_) was complete, with metabolism of riboflavin to flavin mononucleotide and flavin adenine dinucleotide also present. Synthesis of pyridoxine phosphate (vitamin B_6_) from d-erythrose 4-phosphate was almost complete; only a single gene in the pathway, *pdxA*, was missing. An additional blast search did not find this gene, but given that the remainder of the pathway was found, this gene may be missing due to assembly error.

The only pathway where the KAAS and RAST models disagreed was in the metabolism of biotin (vitamin B_7_), with only the RAST model having predicted biotin synthesis. KAAS predicted that most, but not all, of the gene products needed for biotin synthesis were present. Subsequent blastn and tblastx searches using S. praecaptivus genes from the biotin pathway found missing genes, but the genes were not complete. Part of one key gene, *bioF*, was identified on the end of a contig, suggesting that it was present but was not assembled completely. Two other genes (*fabG* and *ynfK*) were not found, but this is again likely due to assembly error. Despite the issues with assembly, the synthesis of biotin does appear similar to that in S. praecaptivus.

Both RAST and KAAS models predicted the conversion of thiamine diphosphate to thiamine phosphate. The conversion is consistent with exogenous scavenging of thiamine diphosphate rather than biosynthesis of thiamine. S. praecaptivus is predicted to have a complete pathway for thiamine synthesis, so we suspected that we may have failed to annotate this in the new endosymbiont genome. Therefore, we searched for a region similar to a genomic region in S. praecaptivus that contains multiple genes involved in thiamine biosynthesis. This region is flanked by two genes that we expected to be conserved between the two species, a 16S rRNA gene and *rpoC*. Using these genes as guides, we identified this region in the Sodalis endosymbiont genome. This region was assembled; however, it appears that the region between these two genes may be degraded in this endosymbiont, with many genes missing (see Fig. S2 in the supplemental material). This includes multiple genes whose products are required for thiamine biosynthesis. Therefore, it appears that this endosymbiont cannot synthesize thiamine.

### Pathogenicity genes in Rickettsia.

From our *de novo* genome assembly, we identified 18 genes possibly associated with virulence in vertebrates in R. rickettsii as identified by Felsheim et al. (34). We found that 5 of these 18 genes were potentially disrupted by mutation based on our assembly. Four of these genes possessed deletions, and one gene contained a frameshift mutation (Table 2). This included an ankyrin repeat-containing gene that was absent or disrupted in R. peacockii and in two attenuated strains of R. rickettsii (34; R. Felsheim, personal communication). A large deletion was found in the ankyrin repeat-containing gene in the fur seal louse-associated Rickettsia bacterium that almost perfectly matched a deletion in the same gene in the attenuated Iowa strain of R. rickettsii.

**TABLE 2:**
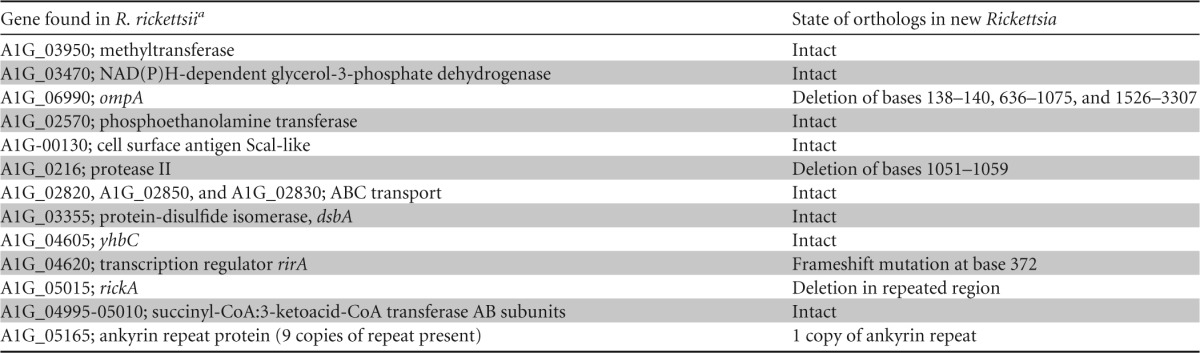
Genes associated with virulence in R. rickettsii SS and their state in the new *Rickettsia* bacterium

a CoA, coenzyme A.

## DISCUSSION

We found that the lice parasitizing the northern fur seals harbor two bacterial species. One was a heritable gammaproteobacterium endosymbiont belonging to the genus Sodalis. The other bacterial associate was identified as an alphaproteobacterium belonging to the genus Rickettsia.

### Sodalis endosymbiont.

Sodalis and closely related endosymbionts form a diverse group of Enterobacteriaceae that have been found in association with tsetse flies, ked flies, rice weevils, solitary bees, stinkbugs, spittlebugs, chewing lice, and, in this instance, blood-sucking lice (49, 62–64, 70–77). This is in addition to the recently described and free-living Sodalis praecaptivus (57, 78). The fur seal louse endosymbiont is more closely related to endosymbionts of chewing lice (that parasitize doves) than to other blood-sucking louse endosymbionts. As chewing lice are moderately closely related to sucking lice, it would seem logical to suspect that Sodalis represents the ancestral symbiont of lice. However, it appears more likely that Sodalis has invaded lice multiple times, likely replacing older endosymbionts.

The species-level relationships of Sodalis were mostly unresolved in our analysis. Smith et al. (49) interpreted this lack of resolution as evidence for repeated recent invasions of chewing lice by Sodalis. Repeated host invasions coupled with accelerated rates of nucleotide substitution in symbiotic species leave the evolutionary relationships of Sodalis difficult to resolve. The resulting star-like patterns (short internal branches with long tips) degrade confidence in the overall relationships between species (49). It does, however, support multiple recent invasions of lice by Sodalis bacteria. As this endosymbiont may have also been recently acquired, it may shed light on how new symbioses have formed with lice. We did not detect an organized bacteriome containing Sodalis, like that harboring “Candidatus Riesia” in human lice (10–12). This less organized storage may represent an early stage of symbiosis between sucking lice and bacteria.

If the seal louse endosymbiont was recently acquired, we might expect to see evidence that its genome is being reduced. By targeting specific orthologous genome regions between the seal louse endosymbiont and the free-living S. preacaptivus genome, we found evidence of gene loss. This degradation has removed redundant functions within the endosymbiont genome and metabolic functions potentially shared with the louse. This includes the loss of genes needed for thiamine and β-alanine synthesis, as well as paralogs involved in flagellar synthesis. The loss of genes appears to be the result of substitutions or small mutations. We saw no evidence that this degradation is the result of insertional sequence proliferation that has been proposed for other Sodalis endosymbionts (e.g., S. pierantonius and S. glossinidius) (79, 80). Future studies should seek to identify pseudogenes throughout the genome for a more global perspective on genome reduction.

**FIG 8:**
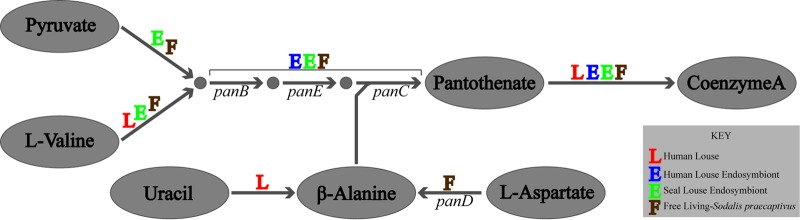
Comparison of pantothenate and CoA biosynthesis by the free-living S. praecaptivus, the fur seal louse Sodalis endosymbiont, the human head louse endosymbiont (“*Ca*. Riesia pediculicola”), and the human louse showing metabolic complementation between endosymbionts through loss of *panD*.

In human lice, endosymbionts (“Candidatus Riesia pediculicola”) have been implicated in provisioning B vitamins that are absent from the louse's diet of blood (2, 3, 24, 61). Given that all sucking lice feed on mammal blood, we suspect that this seal louse endosymbiont may have a similar role. Metabolic models for the seal louse-associated Sodalis endosymbiont suggest it has the capacity to synthesize B vitamins similar to “*Ca*. Riesia” (Table 2). Metabolic complementation between insects and their endosymbionts in the biosynthesis of one of these vitamins, pantothenate, is present in many systems (81). In human lice, the endosymbiont does not appear to be able to synthesize a substrate needed for synthesis of pantothenate, β-alanine (Fig. 8). Instead, this precursor is potentially synthesized by the host and made available to the endosymbiont. In contrast, the free-living S. praecaptivus does retain functionality to produce β-alanine. The S. praecaptivus gene needed for biosynthesis of β-alanine from l-aspartate was identified as a pseudogene in the seal louse endosymbiont. The loss of this gene suggests a dependence on the host for β-alanine by the Sodalis endosymbiont, but the remaining pathway for synthesis of pantothenate appears complete. This is consistent with metabolic complementation between seal lice and the Sodalis endosymbiont. As we were able to identify a candidate pseudogene, this gene may have been disrupted recently.

### Rickettsia.

The fur seal Rickettsia associate was found to belong to the spotted fever species group of Rickettsia. This group includes the arthropod-transmitted pathogenic species R. philipii and R. rickettsii and a symbiotic species, R. peacockii (52, 82, 83). Ixodes ticks are the typical hosts for these Rickettsia species (52). The typical tick vectors for spotted fever group Rickettsia are not known to parasitize the northern fur seals (84–86). This may suggest that lice facilitate transmission of the Rickettsia bacteria, either vertically (from mother to offspring) and/or horizontally between seals they are parasitizing. However, we were unable to detect any evidence of vertical transmission of Rickettsia in the lice. It is possible that the Rickettsia cells were much less abundant than the Sodalis endosymbiont and were not detected or that Rickettsia cells were extracellular and wash away during sample preparation. Taken together, this may suggest this Rickettsia is either a pathogen that is transferred via the alimentary canal between vertebrate hosts or an organism that is accidentally and transiently infecting the lice.

This Rickettsia bacterium has a genome similar to that of its close phylogenetic relative, the pathogenic R. rickettsii, but unlike that of the tick endosymbiont R. peacockii (Fig. 4). The genome of R. peacockii has been extensively rearranged by the presence of a mobile element (34). This rearrangement may be associated with a shift in the life history of this species to obligate endosymbiosis (82). The fact that the seal louse Rickettsia genome is similar to the pathogenic spotted fever group Rickettsia genome could be informative about its life history, suggesting it is a pathogen. However, the genes that may be needed for horizontal transmission by Rickettsia appear to be disrupted. This included five genes identified by Felsheim et al. (34) that appear to be necessary for host invasion and coopting of host cell machinery. This may suggest that this seal louse-associated Rickettsia bacterium is not pathogenic.

Human lice can be infected by Rickettsia prowazekii, and those infections are lethal to the lice due to subsequent rupture of the gut wall (87). This rupture of the gut causes its contents to leak into the hemolymph, giving the lice a visible red color (88). Three additional species of Rickettsia (Rickettsia conorii, R. rickettsii, and Rickettsia *typhii*) have been shown to infect parasitic lice under laboratory conditions (88, 89). These experimental infections also resulted in rupture of the louse gut similar to that which occurs with R. prowazekii infections (88, 89). Despite the presence of a Rickettsia species in fur seal lice, we saw no evidence of the pathology in any of the lice during visual examination. This observation along with potential loss-of-function mutations in genes important to pathogenicity could suggest this is an attenuated strain of Rickettsia. However, these conclusions are tentative, as we were unable to find evidence of vertical transmission and unable to confirm the gene deletions using PCR (available specimens were consumed in genome sequencing and *in situ* characterization). Future investigation should focus on obtaining additional specimens for confirmation of deletions in these genes and verifying the organism's phenotype in culture.

## Supplementary Material

Supplemental material

## References

[1] Grimaldi D, Engel MS 2005 Evolution of the insects. Cambridge University Press, Cambridge, United Kingdom.

[2] Puchta O 1955 Experimentelle untersuchungen uber die bedeutung der symbiose der kleiderlaus Pediculus vestimenti Burm. Z Parasitenkd 17:1. 13248062 10.1007/BF00260226

[3] Perotti MA, Kirkness EF, Reed DL, Braig HR 2009 Endosymbionts of lice, p 205–219. *In* Bourtzis K, Miller TA (ed), Insect symbiosis, vol 3 CRC Press, Boca Raton, FL.

[4] Ries E 1931 Die symbiose der lause und federlinge. Z Morphol Okol Tiere 20:233–67. doi:10.1007/BF00444101.

[5] Ries E 1932 Die prozesse der eibildung und des eiwachstums bei Pediculiden und Mallophagen. Z Zellforsch Mikrosk Anat 16:314–388. doi:10.1007/BF00390954.

[6] Aschner M, Ries E 1933 Das verhalten der kleiderlaus bei ausschaltung iher symbioten. Z Morphol Okol Tiere 26:529–590. doi:10.1007/BF00446386.

[7] Ries E 1933 Endosymbiose und parasitismus. Z Parasitenkd 6:339–349. doi:10.1007/BF02121953.

[8] Ries E, van Weel PB 1934 Die eibildung der kleiderlaus, untersucht an lebenden, vital gefarbten und fixierten praparaten. Z Zellforsch Mikrosk Anat 20:565–618. doi:10.1007/BF00533771.

[9] Ries E 1935 Uber den sinn der erblichen insektensymbiose. Naturwissenschaften 23:744–749. doi:10.1007/BF01494010.

[10] Buchner P 1965 Endosymbiosis of animals with plant microorganisms. Interscience, New York, NY.

[11] Sasaki-Fukatsu K, Koga R, Nikoh N, Yoshizawa K, Kasai S, Mihara M, Kobayashi M, Tomita T, Fukatsu T 2006 Symbiotic bacteria associated with stomach discs of human lice. Appl Environ Microbiol 72:7349–7352. doi:10.1128/AEM.01429-06. 16950915 PMC1636134

[12] Perotti MA, Allen JM, Reed DL, Braig HR 2007 Host-symbiont interactions of the primary endosymbiont of human head and body lice. FASEB J 21:1058–1066. doi:10.1096/fj.06-6808com. 17227954

[13] Allen JM, Reed DL, Perotti MA, Braig HR 2007 Evolutionary relationships of “Candidatus Riesia spp.,” endosymbiotic Enterobacteriaceae living within hematophagous primate lice. Appl Environ Microbiol 73:1659–1664. doi:10.1128/AEM.01877-06. 17220259 PMC1828778

[14] Allen JM, Light JE, Perotti MA, Braig HR, Reed DL 2009 Mutational meltdown in primary endosymbionts: selection limits Muller's ratchet. PLoS One 4:e4969. doi:10.1371/journal.pone.0004969. 19305500 PMC2654755

[15] Hypsa V, Krizek J 2007 Molecular evidence for polyphyletic origin of the primary symbionts of sucking lice (Phthirpatera: Anoplura). Microb Ecol 54:242–251. doi:10.1007/s00248-006-9194-x. 17345136

[16] Fukatsu T, Hosokawa T, Koga R, Nikoh N, Kato T, Hayama S, Takefushi H, Tanaka I 2009 Intestinal endocellular symbiotic bacterium of the macaque louse Pedicinus obtusus: distinct endosymbiont origins in anthropoid primate lice and the Old World monkey louse. Appl Environ Microbiol 75:3796–3799. doi:10.1128/AEM.00226-09. 19304816 PMC2687293

[17] Nováková E, Hypsa V, Moran NA 2009 Arsenophonus, an emerging clade of intracellular symbionts with a broad host distribution. BMC Microbiol 9:143. doi:10.1186/1471-2180-9-143. 19619300 PMC2724383

[18] Eberle MW, McLean DL 1982 Initiation and orientation of the symbiote migration in the human body louse Pediculus humans L. J Insect Physiol 28:417–422. doi:10.1016/0022-1910(82)90068-3.

[19] Eberle MW, McLean DL 1983 Observation of symbiote migration in human body lice with scanning and transmission electron microscopy. Can J Microbiol 29:755–762. doi:10.1139/m83-123. 6413046

[20] Boyd BM, Reed DL 2012 Taxonomy of lice and their endosymbiotic bacteria in the postgenomic era. Clin Microbiol Infect 18:324–331. doi:10.1111/j.1469-0691.2012.03782.x. 22429457

[21] Durden LA, Musser GG 1994 The mammalian hosts of sucking lice (Anoplura) of the world: a host-parasite list. J Vector Ecol 19:130–168.

[22] Nowak RM 1991 Walker's mammals of the world, 5th ed, vol 11 Johns Hopkins University Press, Baltimore, MD.

[23] Kim KC 1971 The sucking lice (Anoplura: Echinophthiriidae) of the northern fur seal: descriptions and morphological adaptation. Ann Entomol Soc Am 64:280–292. doi:10.1093/aesa/64.1.280.

[24] Boyd BM, Allen JM, de Crecy-Lagard V, Reed DL 2014 Genome sequence of Candidatus Riesia pediculischaeffi endosymbiont of chimpanzee lice, and genomic comparison of recently acquired endosymbionts from human and chimpanzee lice. G3 (Bethesda) 4:2189–2195. doi:10.1534/g3.114.012567. 25213693 PMC4232544

[25] Simpson JT, Wong K, Jackman SD, Schein JE, Jones SJM, Birol I 2009 ABySS: a parallel assembler for short read sequence data. Genome Res 19:1117–1123. doi:10.1101/gr.089532.108. 19251739 PMC2694472

[26] Altschul SF, Gish W, Miller W, Myers EW, Lipman DJ 1990 Basic local alignment search tool. J Mol Biol 215:403–410. doi:10.1016/S0022-2836(05)80360-2. 2231712

[27] Langmead B, Saizberg S 2012 Fast gapped-read alignment with Bowtie 2. Nat Methods 9:357–359. doi:10.1038/nmeth.1923. 22388286 PMC3322381

[28] Overbeek R, Begley T, Butler RM, Choudhuri JV, Chuang HY, Cohoon M, de Crecy-Lagard V, Diaz N, Disz T, Edwaards R, Fonstein M, Frank ED, Gerdes S, Glass EM, Goesmann A, Hanson A, Iwata-Reuyl D, Jensen R, Jamshidi N, Krause L, Kubal M, Larsen N, Linke B, McHardy AC, Meyer F, Neuweger H, Olsen G, Olsen R, Osterman A, Portnoy V, Pusch GD, Rodionov DA, Ruckert C, Steiner J, Stevens R, Thiele I, Vassieva O, Ye Y, Zagnitko O, Vonstein V 2005 The subsystems approach to genome annotation and its use in the project to annotate 1,000 genomes. Nucleic Acids Res 33:5691–5702. doi:10.1093/nar/gki866. 16214803 PMC1251668

[29] Aziz RK, Bartels D, Best AA, DeJongh M, Disz T, Edwards RA, Formsma K, Gerdes S, Glass EM, Kubal M, Meyer F, Olsen GJ, Olson R, Osterman AL, Overbeek RA, McNeil LK, Paarmann D, Paczian T, Parrello B, Pusch C, Reich C, Stevens R, Vassieva O, Vonstein V, Wilke A, Zagnitko O 2008 The RAST server: rapid annotations using subsystems technology. BMC Genomics 9:75. doi:10.1186/1471-2164-9-75. 18261238 PMC2265698

[30] Soderlund C, Nelson W, Shoemaker A, Paterson A 2006 SyMAP: a system for discovering and viewing syntenic regions of FPC maps. Genome Res 16:1159–1168. doi:10.1101/gr.5396706. 16951135 PMC1557773

[31] Soderlund C, Bomboff M, Nelson W 2011 SyMAP: a turnkey synteny system with applications to plant genomes. Nucleic Acids Res 39:e68. doi:10.1093/nar/gkr123. 21398631 PMC3105427

[32] Li H, Handsaker B, Wysoker A, Fennell T, Ruan J, Homer N, Marth G, Abecasis G, Durbin R, 1000 Genome Project Data Processing Subgroup 2009 The sequence alignment/map (SAM) format and SAMtools. Bioinformatics 25:2078–2079. 19505943 10.1093/bioinformatics/btp352 PMC2723002

[33] Li H 2011 A statistical framework for SNP calling, mutation discovery, association mapping and population genetical parameter estimation from sequencing data. Bioinformatics 27:2987–2993. doi:10.1093/bioinformatics/btr509. 21903627 PMC3198575

[34] Felsheim RF, Kurtti TJ, Munderloh UG 2009 Genome sequence of the endosymbiont Rickettsia peacockii and comparison with virulent Rickettsia rickettsii: identification of virulence factors. PLoS One 4:e8361. doi:10.1371/journal.pone.0008361. 20027221 PMC2791219

[35] Edgar RC 2004 MUSCLE: multiple sequence alignment with high accuracy and high throughput. Nucleic Acids Res 32:1792–1797. doi:10.1093/nar/gkh340. 15034147 PMC390337

[36] Lyons E, Freeling M 2008 How to usefully compare homologous plant genes and chromosomes as DNA sequences. Plant J 53:661–673. doi:10.1111/j.1365-313X.2007.03326.x. 18269575

[37] Lyons E, Pendersen B, Kane J, Freeling M 2008 The value of nonmodel genomes and an example using SynMap with CoGe to dissect the hexaploidy that predates rosids. Trop Plant Biol 1:181–190. doi:10.1007/s12042-008-9017-y.

[38] Allen JM, Huang DI, Cronk QC, Johnson KP 2015 aTRAM: automated target restricted assembly method—a fast method for assembling loci across divergent taxa from next-generation sequencing data. BMC Bioinformatics 16:98. doi:10.1186/s12859-015-0515-2. 25887972 PMC4380108

[39] McNeil LK, Reich C, Aziz RK, Bartels D, Cohoon M, Disz T, Edwards RA, Gerdes S, Hwang K, Kubal M, Margaryan GR, Meyer F, Mihalo W, Olsen GJ, Olson R, Osterman A, Paarmann D, Paczian T, Parrello B, Pusch GD, Rodionov DA, Shi X, Vassieva O, Vonstein V, Zangnitko O, Xia F, Zinner K, Overbeek R, Stevens R 2007 The National Microbial Pathogen Database Resource (NMPDR): a genomics platform based on subsystem annotation. Nucleic Acids Res 35:D347–D353. doi:10.1093/nar/gkl947. 17145713 PMC1751540

[40] Moriya Y, Itoh M, Okuda S, Yoshizawa AC, Kanehisa M 2007 KAAS: an automatic genome annotation and pathway reconstruction server. Nucleic Acids Res 35:W182–W185. doi:10.1093/nar/gkm321. 17526522 PMC1933193

[41] Akiyama Y, Goto S, Uchiyama I, Kanehisa M 1995 WebDBGET: an integrated database retrieval system which provides hyper-links among related entries. 2nd Meeting on the Interconnection of Molecular Biology Databases.

[42] Goto S, Akiyama Y, Kanehisa M 1995 LinkDB: a database of cross links between molecular biology databases. 2nd Meeting on the Interconnection of Molecular Biology Databases.

[43] Fujibuchi W, Goto S, Migimatsu H, Uchiyama I, Ogiwara A, Akiyama Y, Kanehisa M 1998 DBGET/LinkDB: an integrated database retrieval system. Pac Symp Biocomput 1998:683–694. 9697222

[44] Kanehisa M 1997 Linking databases and organisms: GenomeNet resources in Japan. Trends Biochem Sci 22:442–444. doi:10.1016/S0968-0004(97)01130-4. 9397687

[45] Kanehisa M, Goto S 2000 KEGG: Kyoto Encyclopedia of Genes and Genomes. Nucleic Acids Res 28:27–30. doi:10.1093/nar/28.1.27. 10592173 PMC102409

[46] Kanehisa M, Goto S, Sato Y, Kawashima M, Furumichi M, Tanabe M 2014 Data, information, knowledge and principle: back to metabolism in KEGG. Nucleic Acids Res 42:D199–D205. doi:10.1093/nar/gkt1076. 24214961 PMC3965122

[47] Welch MD, Rosenblatt J, Skoble J, Portnoy DA, Mitchison TJ 1998 Interaction of human Arp2/3 complex and the Listeria monocytogenes ActA protein in filament nucleation. Science 281:105–108. doi:10.1126/science.281.5373.105. 9651243

[48] Kriventseva EV, Tegenfeldt F, Petty TJ, Waterhouse RM, Simao FA, Pozdnyakov IA, Ioannidis P, Zdobnov EM 2015 OrthoDB v8: update of the hierarchical catalog of orthologs and the underlying free software. Nucleic Acids Res 43:D250–D256. doi:10.1093/nar/gku1220. 25428351 PMC4383991

[49] Smith WA, Oakeson KF, Johnson KP, Reed DL, Carter T, Smith KL, Koga R, Fukatsu T, Clayton DH, Dale C 2013 Phylogenetic analysis of symbionts in feather-feeding lice of the genus Columbicola: evidence of repeated symbiont replacements. BMC Evol Biol 13:109. doi:10.1186/1471-2148-13-109. 23725492 PMC3724504

[50] Lanfear R, Calcott R, Ho SYW, Guidon S 2012 PartitionFinder: combined selection of partitioning schemes and substitution models for phylogenetic analysis. Mol Biol Evol 29:1695–1701. doi:10.1093/molbev/mss020. 22319168

[51] Stamatakis A 2006 RAxML-VI-HPC: maximum likelihood-based phylogenetic analyses with thousands of taxa and mixed models. Bioinformatics 22:2688–2690. doi:10.1093/bioinformatics/btl446. 16928733

[52] Weinert LA, Werren JH, Aebi A, Stone GN, Jiggins FM 2009 Evolution and diversity of Rickettsia bacteria. BMC Biol 7:6. doi:10.1186/1741-7007-7-6. 19187530 PMC2662801

[53] Lane DJ 1991 16S/23S rRNA sequencing, p 115–175. *In* Stackebrandt E, Goodfellow M (ed), Bacterial systematics. Wiley, Chichester, United Kingdom.

[54] Fukatsu T 1999 Acetone preservation: a practical technique for molecular analysis. Mol Ecol 8:1935–1945. doi:10.1046/j.1365-294x.1999.00795.x. 10620236

[55] Quast C, Pruesse E, Yilman P, Gerken J, Schweer T, Yarza P, Peplies J, Glockner FO 2013 The SILVA ribosomal RNA gene database project: improved data processing and web-based tools. Nucleic Acids Res 41:D590–D596. doi:10.1093/nar/gks1219. 23193283 PMC3531112

[56] Loy A, Maixner F, Wagner M, Horn M 2007 probeBase: an online resource for rRNA-targeted oligonucleotide probes—new features 2007. Nucleic Acids Res 35:D800–D804. doi:10.1093/nar/gkl856. 17099228 PMC1669758

[57] Clayton AL, Oakeson KF, Gutin M, Pontes A, Dunn DM, Niederhausern ACV, Weiss RB, Fisher M, Dale C 2012 A novel human-infection-derived bacterium provides insights into the evolutionary origins of mutualistic insect-bacterial symbiosis. PLoS Genet 8:e1002990. doi:10.1371/journal.pgen.1002990. 23166503 PMC3499248

[58] Oakeson KF, Gil R, Clayton AL, Dunn DM, von Niderhausern AC, Hamil C, Aoyagi A, Duval B, Baca A, Silva FJ, Vallier A, Jackson DG, Latorre A, Weiss RB, Heddi A, Moya A, Dale C 2014 Genome degeneration and adaptation in a nascent stage of symbiosis. Genome Biol Evol 6:76–93. doi:10.1093/gbe/evt210. 24407854 PMC3914690

[59] Koga R, Moran NA 2014 Swapping symbionts in spittlebugs: evolutionary replacement of a reduced genome symbiont. ISME J 8:1237–1246. doi:10.1038/ismej.2013.235. 24401857 PMC4030230

[60] Toh H, Weiss BL, Perkin SAH, Yamashita A, Oshima K, Hattori M, Aksoy S 2006 Masssive genome erosion and functional adaptations provide insights into the symbiotic lifestyle of Sodalis glossinidius in the tsetse host. Genome Res 16:149–156. 16365377 10.1101/gr.4106106 PMC1361709

[61] Kirkness EF, Haas BJ, Sun WL, Braig HR, Perotti MA, Clark JM, Lee SH, Robertson HM, Kennedy RC, Elhaik E, Gerlack D, Kriventseva EV, Elsik CG, Graur D, Hill CA, Veenstra JA, Walenz B, Tubio JMC, Ribeiro JMC, Rozas J, Johnston JS, Reese JT, Popadic A, Tojo M, Raoult D, Reed DL, Tomoyasu Y, Krause E, Mittapalli O, Margam VM, Li HM, Meyer JM, Johnson RM, Romero-Severson J, VanZee JP, Alvarez-Ponce D, Vieira FG, Aguade M, Guirao-Rico S, Anzola JM, Yoon KS, Strycharz JP, Unger MF, Christley S, Lobo NF, Seufferheld MJ, Wang NK, Dasch GA, Struchiner CJ, Madey G, 2010 Genome sequence of the human body louse and its primary endosymbiont provide insights into the permanent parasitic lifestyle. Proc Natl Acad Sci U S A 107:12168–12173. doi:10.1073/pnas.1003379107. 20566863 PMC2901460

[62] Aksoy S, Chen X, Hypsa V 1997 Phylogeny and potential transmission routes of midgut-associated endosymbionts of tsetse (Diptera: Glossinidae). Insect Mol Biol 6:183–190. doi:10.1111/j.1365-2583.1997.tb00086.x. 9099582

[63] Fukatsu T, Koga R, Smith WA, Tanaka K, Nikoh N, Sasaki-Fukatsu K, Yoshizawa K, Cale C, Clayton DH 2007 Bacterial endosymbiont of the slender pigeon louse, Columbicola columbae, allied to endosymbionts of grain weevils and tsetse flies. Appl Environ Microbiol 73:6660–6668. doi:10.1128/AEM.01131-07. 17766458 PMC2075037

[64] Kaiwa N, Hosokawa T, Kikuchi Y, Hikoh N, Meng XY, Kimura N, Ito M, Fukatsu T 2010 Primary gut symbionts and secondary, Sodalis-allied symbiont of the scutellerid stinkbug Cantao ocellatus. Appl Environ Microbiol 76:3486–3494. doi:10.1128/AEM.00421-10. 20400564 PMC2876435

[65] Attardo GM, Lohs C, Heddi A, Alam UH, Yildirim S, Aksoy S 2008 Analysis of milk gland structure and functions in Glossina morsitans: milk protein production, symbiont populations and fecundity. J Insect Physiol 54:1236–1242. doi:10.1016/j.jinsphys.2008.06.008. 18647605 PMC2613686

[66] Bright M, Bulgheresi S 2010 A complex journey: transmission of microbial symbionts. Nat Rev Microbiol 8:218–230. doi:10.1038/nrmicro2262. 20157340 PMC2967712

[67] Balmand S, Lohs C, Aksoy S, Heddi A 2013 Tissue distribution and transmission routes for the tsetse fly endosymbionts. J Invertebr Pathol 112:S116–S122. doi:10.1016/j.jip.2012.04.002. 22537833 PMC3772537

[68] De Vooght L, Caljon G, Van Hees J, Den Abbeele JV 2015 Paternal transmission of a secondary symbiont during mating in the viviparous tsetse fly. Mol Biol Evol 32:1977–1980. doi:10.1093/molbev/msv077. 25851957 PMC4833065

[69] Lambrechts A, Gevaert K, Cossar P, Vandekerckhove J, Van Troys M 2008 Listeria comet tails: the actin-based motility machinery at work. Trends Cell Biol 18:220–227. doi:10.1016/j.tcb.2008.03.001. 18396046

[70] Heddi A, Charles H, Khatchadourian C, Bonnot G, Nardon P 1998 Molecular characterization of the principal symbiotic bacteria of the weevil Sitophilus oryzae: a peculiar G+C content of endocytobiotic DNA. J Mol Evol 47:52–61. doi:10.1007/PL00006362. 9664696

[71] Dale C, Maudlin I 1999 Sodalis gen. nov. and Sodalis glossinidius sp. nov., a microaerophilic secondary endosymbiont of the tsetse fly Glossina morsitans morsitans. Int J Syst Bacteriol 49:267–275. doi:10.1099/00207713-49-1-267. 10028272

[72] Nováková E, Hypsa V 2007 A new Sodalis lineage from bloodsucking fly Caterina melbae (Diptera: Hippoboscoidea) originated independently of the tsetse fly symbiont Sodalis glossinidius. FEMS Microbiol Lett 269:131–135. doi:10.1111/j.1574-6968.2006.00620.x. 17227456

[73] Chrudimský T, Husnik F, Novakova E, Hypsa V 2012 Candidatus Sodalis melophagi sp. nov.: phylogenetically independent comparative model to the tsetse fly symbiont Sodalis glossinidius. PLoS One 7:e40354. doi:10.1371/journal.pone.0040354. 22815743 PMC3398932

[74] Koga R, Bennet GM, Cryan JR, Moran NA 2013 Evolutionary replacement of obligate symbionts in an ancient and diverse insect lineate. Environ Microbiol 15:2073–2081. doi:10.1111/1462-2920.12121. 23574391

[75] Matsuura Y, Hosokawa T, Serracin M, Tulgetske GM, Miller TA, Fukatsu T 2014 Bacterial symbionts of devastating coffee plant pest, the stinkbug Antestiopsis thunbergii (Hemiptera: Pentatomidae). Appl Environ Mircobiol 80:3769–3775. doi:10.1128/AEM.00554-14. PMC4054137 24727277

[76] Saeed A 2014 Characterizing the maternally inherited endosymbiont of solitary bees. Theses and Dissertations–Entomology, University of Kentucky http://uknowledge.uky.edu/entomology_etds/10/.

[77] Hosokawa T, Kaiwa N, Matsuura Y, Kikuchi Y, Fukatsu T 2015 Infection prevalence of Sodalis symbionts among stinkbugs. Zool Lett 1:5. doi:10.1186/s40851-014-0009-5. PMC4604117 26605050

[78] Chari A, Oakeson KF, Enomoto S, Jackson DG, Fisher MA, Dale C 2015 Phenotypic characterization of Sodalis praecaptivus sp. nov., a close noninsect associated member of Sodalis-allied lineage of insect endosymbionts. Int J Syst Evol Microbiol 65:1400–1405. doi:10.1099/ijs.0.000091. 25782768 PMC4635462

[79] Gil R, Belda E, Gosalbes MJ, Delaye L, Vallier A, Vincent-Monegat C, Heddi A, Silva FJ, Moya A, Latorre A 2008 Massive presence of insertion sequences in the genome of SOPE, the primary endosymbiont of the rice weevil Sitophilus oryzae. Int Microbiol 11:41–48. 18683631

[80] Belda E, Moya A, Bentley S, Silva FJ 2010 Mobile genetic element proliferation and gene inactivation impact over the genome structure and metabolic capabilities of Sodalis glossinidius, the secondary endosymbiont of tsetse flies. BMC Genomics 11:449. doi:10.1186/1471-2164-11-449. 20649993 PMC3091646

[81] Wilson ACC, Duncan RP 2015 Signatures of host/symbiont genome coevolution in insect nutritional endosymbiosis. Proc Natl Acad Sci U S A 112:10255–10261. doi:10.1073/pnas.1423305112. 26039986 PMC4547219

[82] Niebylski ML, Schrumpt ME, Burgdorfer W, Fisher ER, Gage KL, Schwan TG 1997 Rickettsia peacockii sp. nov., a new species infecting wood ticks, Dermacentor andersoni, in western Montana. Int J Syst Bacteriol 47:446–452. doi:10.1099/00207713-47-2-446. 9103635

[83] Burgdorfer W, Hayes SF, Maves AJ 1981 Nonpathogenic rickettsiae in Dermacentor andersoni: a limiting factor for the distribution of Rickettsia rickettsii, p 585–594. *In* Burgdorfer W, Anaker RL (ed), Rickettsiae and rickettsial diseases. Academic Press, New York, NY.

[84] Ward BT 1921 Alaska fishery and fur-seal industries in 1920. *In* Appendix VI of Doc 909 of the Report to the U.S. Commissioner of Fisheries for 1921.

[85] Furman DP, Loomis EC 1984 The ticks of California (Acari: Ixodida). Bull Calif Insect Surv, vol 25 University of California Press, Berkeley, CA.

[86] Osborne H 1899 Insects of the Pribilof Islands. IX. Acarina, p 553–554. *In* Jordan DS, Stejneger K, Lucas FA, Moser JF, Townsend CH, Clark GA, Murray J (ed), Fur seals and fur-seal islands, North Pacific Ocean, part 3. US Government Printing Office, Washington, DC.

[87] Houhamdi L, Fournier PE, Fang R, Raoult D 2003 An experimental model of human body louse infection with Rickettsia typhi. Ann N Y Acad Sci 990:617–627. doi:10.1111/j.1749-6632.2003.tb07436.x. 12860699

[88] Raoult D, Roux V 1999 The body louse as a vector of reemerging human diseases. Clin Infect Dis 29:888–911. doi:10.1086/520454. 10589908

[89] Houhamdi L, Raoult D 2006 Experimentally infected human body lice (Pediculus humanus humanus) as vectors of Rickettsia rickettsii and Rickettsia conorii in a rabbit model. Am J Trop Med Hyg 74:521–525. 16606977

