# Supplementary material for: Two Bacterial Genera, Sodalis and Rickettsia, Associated with the Seal Louse Proechinophthirus fluctus (Phthiraptera: Anoplura)
Source: Appl Environ Microbiol. 2016 May 16;82(11):3185–97. doi: 10.1128/AEM.00282-16 (PMC4959230; doi:10.1128/AEM.00282-16)
Supplement: Supplemental material [file supp_82_11_3185__index.html]

Supplemental material 

# Two Bacterial Genera, Sodalis and Rickettsia, Associated with the Seal Louse Proechinophthirus fluctus (Phthiraptera: Anoplura)

## Supplemental material

- Supplemental file 1 -

  Most likely tree based on maximum likelihood analysis of *groEL* sequences from *Sodalis* bacteria and allied endosymbionts, representative *Enterobacteriaceae*, and outgroup taxa from *Pasteurellaceae* (Fig. S1); alignment between the *S. praecaptivus* genome region containing genes encoding products involved in thiamin biosynthesis and seal louse endosymbiont genome contig\_2869 (Fig. S2); predicted genes necessary for flagellum construction and B vitamin biosynthesis (Table S1); gene counts in RAST function categories (Table S2); partial sequences of *coaA* generated using aTRAM software and a translated sequence of *panD* pseudogene identified manually (Supplemental Data).

  PDF, 2.1M
