# Supplementary material for: Two Bacterial Genera, Sodalis and Rickettsia, Associated with the Seal Louse Proechinophthirus fluctus (Phthiraptera: Anoplura)
Source: Appl Environ Microbiol. 2016 May 16;82(11):3185–97. doi: 10.1128/AEM.00282-16 (PMC4959230; doi:10.1128/AEM.00282-16)
Supplement: Supplemental material [file AEM.00282-16_zam999117155so1.pdf]

Supplementary Material

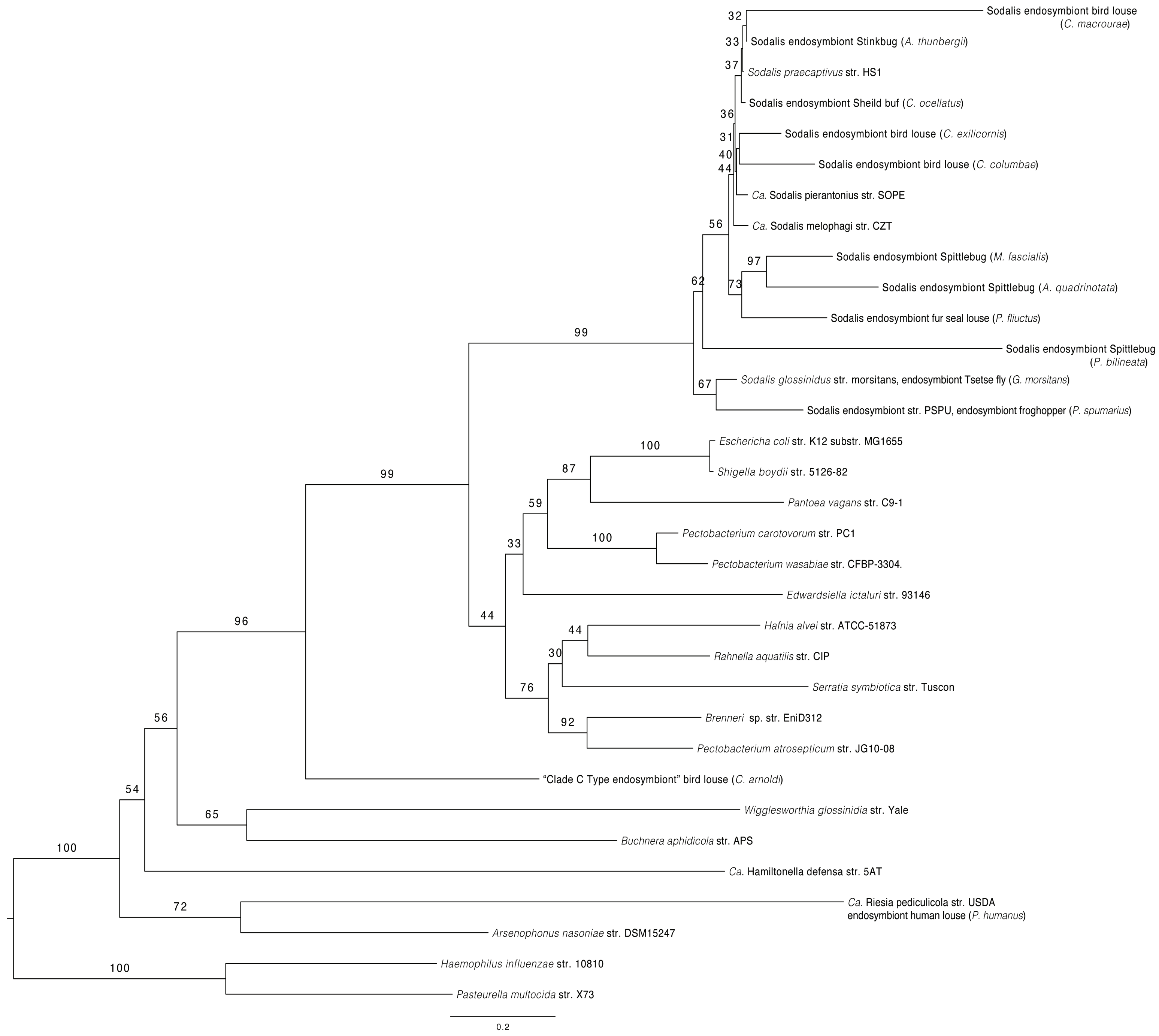

Figure Supplementary-1: Most likely tree based on maximum likelihood analysis of *groEL* sequences from *Sodalis* and allied endosymbionts, representative Enterbacteriaceae, and outgroup taxa from Pasteurellaceae. Numbers at nodes indicate percent of 1000 bootstrap replicates. Vertical bar delineates the *Sodalis* group of bacteria and arrows indicate louse endosymbionts. The Clade-C type endosymbiont from bird lice (*Columbicola arnoldi*) represents an Enterobacteriaceae endosymbiont distantly related to *Sodalis* described by Smith et al. (1). Other bird louse endosymbionts were described by Smith et al. (1) as belonging to Clade-A from *Columbicola*, a *Sodalis* clade.

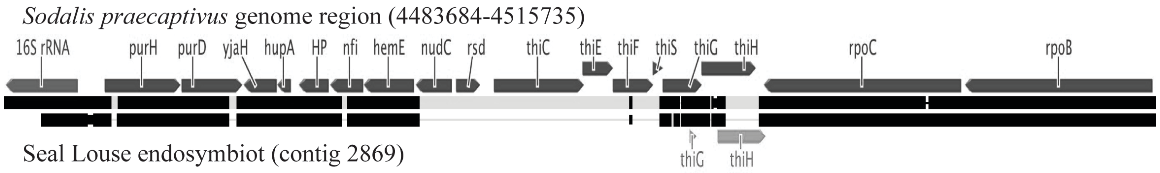

Figure Supplementary-2: Alignment between *S. praecaptivus* genome region containing genes encoding products involved in thiamin biosynthesis (*thiH, thiG, thiS, thiF,* and *thiC*) and seal louse endosymbiont genome contig_2869. Alignment conducted using Muscle (2) with aligned regions highlighted in black and predicted genes annotated in gray and labeled. Image produced using Geneious (Biomaters, www.geneious.com).

Table Supplementary-1: Predicted genes necessary for flagella construciton and B-vitamin biosynthesis

Organism	Role	Gene	Contig	Start-Stop
Sodalis endosymbiont	Flagella	flhD	2851	25158-24814
Sodalis endosymbiont	Flagella	flhC	2851	24809-24219
Sodalis endosymbiont	Flagella	motA	2851	24120-23224
Sodalis endosymbiont	Flagella	motB	2851	23227-22187
Sodalis endosymbiont	Flagella	flhB	2851	21994-20827
Sodalis endosymbiont	Flagella	flhA	2851	20846-18750
Sodalis endosymbiont	Flagella	flgN	2851	17917-18360
Sodalis endosymbiont	Flagella	flgA	2851	16826-17553
Sodalis endosymbiont	Flagella	flgB	2851	16627-16214
Sodalis endosymbiont	Flagella	flgC	2851	16207-15803
Sodalis endosymbiont	Flagella	flgD	2851	15791-14972
Sodalis endosymbiont	Flagella	flgE	2851	14920-13682
Sodalis endosymbiont	Flagella	flgF	2851	13669-12905
Sodalis endosymbiont	Flagella	flgG	2851	12884-12102
Sodalis endosymbiont	Flagella	flgH	2851	12036-11278
Sodalis endosymbiont	Flagella	flgI	2851	11247-10150
Sodalis endosymbiont	Flagella	flgJ	2851	10150-9197
Sodalis endosymbiont	Flagella	flgK	2851	9088-7421
Sodalis endosymbiont	Flagella	flgL	2851	7364-6426
Sodalis endosymbiont	Flagella	flgM-pseudogene	2851	17599-17878
Sodalis endosymbiont	Flagella	fliR	2851	5577-6362
Sodalis endosymbiont	Flagella	fliQ	2851	5301-5571
Sodalis endosymbiont	Flagella	fliP	2851	4481-5282
Sodalis endosymbiont	Flagella	fliO	2851	4082-4456
Sodalis endosymbiont	Flagella	fliN	2851	3665-4081
Sodalis endosymbiont	Flagella	fliM	2851	2644-3672
Sodalis endosymbiont	Flagella	fliK	2851	799-2028
Sodalis endosymbiont	Flagella	fliJ	2851	353-802
Sodalis endosymbiont	Flagella	fliI-part	2851	1-342
Sodalis endosymbiont	Flagella	fliI-part	2871	213-1
Sodalis endosymbiont	Flagella	fliH	2871	896-210
Sodalis endosymbiont	Flagella	fliG	2871	1884-889
Sodalis endosymbiont	Flagella	fliF	2871	3572-1881
Sodalis endosymbiont	Flagella	fliE	2871	3817-4140
Sodalis endosymbiont	Flagella	fliT-pseudogene	2871	4788-4595
Sodalis endosymbiont	Flagella	fliS	2871	5190-4801
Sodalis endosymbiont	Flagella	fliD	2871	6702-5257
Sodalis endosymbiont	Flagella	fliC1	2871	7221-8513
Sodalis endosymbiont	Flagella	flgM2	2885	9650-9946
Sodalis endosymbiont	Pantothenate-CoA synthesis	ilvG	2814	7291-8244
Sodalis endosymbiont	Pantothenate-CoA synthesis	ilvM	2814	8164-8420
Sodalis endosymbiont	Pantothenate-CoA synthesis	ilvH	2845	4570-5068
Sodalis endosymbiont	Pantothenate-CoA synthesis	ilvI	2845	2824-4565
Sodalis endosymbiont	Pantothenate-CoA synthesis	ilvE	2814	8443-9405
Sodalis endosymbiont	Pantothenate-CoA synthesis	ilvD	2814	9485-11340
Sodalis endosymbiont	Pantothenate-CoA synthesis	ilvC	2814	13594-15089
Sodalis endosymbiont	Pantothenate-CoA synthesis	panE	2876	10192-9281
Sodalis endosymbiont	Pantothenate-CoA synthesis	panB	2798	321-1116
Sodalis endosymbiont	Pantothenate-CoA synthesis	panC	2798	1128-1982
Sodalis endosymbiont	Pantothenate-CoA synthesis	panD-pseudogene	2798	2002-2395
Sodalis endosymbiont	Pantothenate-CoA synthesis	coaA-part	see supplement	
Sodalis endosymbiont	Pantothenate-CoA synthesis	dfp-part	2858	1-717
Sodalis endosymbiont	Pantothenate-CoA synthesis	dfp-part	2801	111-9
Sodalis endosymbiont	Pantothenate-CoA synthesis	coaD	2801	3336-2866
Sodalis endosymbiont	Pantothenate-CoA synthesis	coaE	2798	35140-35764
Sodalis endosymbiont	Folate synthesis	pabAB	2882	51622-50261
Sodalis endosymbiont	Folate synthesis	pabAB	2802	19741-20322
Sodalis endosymbiont	Folate synthesis	pabC	2887	68897-69694
Sodalis endosymbiont	Folate synthesis	folP	2870	8585-8779
Sodalis endosymbiont	Folate synthesis	folC-part	2832	731-1
Sodalis endosymbiont	Folate synthesis	folA	2870	73094-73732
Sodalis endosymbiont	Riboflavin synthesis	ribA	2839	2197-2739
Sodalis endosymbiont	Riboflavin synthesis	ribDR	2876	188-328
Sodalis endosymbiont	Riboflavin synthesis	ribB	2884	8873-3217
Sodalis endosymbiont	Riboflavin synthesis	ribH	2876	355-885
Sodalis endosymbiont	Riboflavin synthesis	ribE	2817	115875-116570
Sodalis endosymbiont	Riboflavin synthesis	ribF	2870	62056-62943
Sodalis endosymbiont	Riboflavin synthesis	EPD	2839	43374-44372
Sodalis endosymbiont	Riboflavin synthesis	pdxB	2832	5246-4749
Sodalis endosymbiont	Riboflavin synthesis	serC	2826	8147-7812
Sodalis endosymbiont	Riboflavin synthesis	pdxA	missing	
Sodalis endosymbiont	Riboflavin synthesis	pdxJ	2881	4542-4123
Sodalis endosymbiont	Riboflavin synthesis	pdxP	missing	
Sodalis endosymbiont	Biotin synthesis	fabB-part	2848	1-203
Sodalis endosymbiont	Biotin synthesis	fabB-part	2851	3227-2947
Sodalis endosymbiont	Biotin synthesis	fabF	2851	79176-77926
Sodalis endosymbiont	Biotin synthesis	fabG	missing	
Sodalis endosymbiont	Biotin synthesis	fabZ	2856	25419-25844
Sodalis endosymbiont	Biotin synthesis	fabI	2810	825-37
Sodalis endosymbiont	Biotin synthesis	bioH	2833	9287-8697
Sodalis endosymbiont	Biotin synthesis	bioF-part	2838	195-1
Sodalis endosymbiont	Biotin synthesis	bioA	2838	1493-2193
Sodalis endosymbiont	Biotin synthesis	ynfK	missing	
Sodalis endosymbiont	Biotin synthesis	bioB	2838	1223-405
Sodalis endosymbiont	Nicotinamide synthesis	nadRD	2870	45582-44281
Sodalis endosymbiont	Nicotinamide synthesis	ppnK	2881	19571-20497
Sodalis endosymbiont	Thiamin	TPK	2876	1807-2049
Sodalis endosymbiont	Thiamin	thiG	2869	8265-8390
Sodalis endosymbiont	Thiamin	thiH	2869	8827-9114

Table Supplementary-2. Gene counts in RAST (3-4) function categories

RAST fuctional groups	Seal Louse symbiont	*S. glossinidius*	*S. praecaptivus*	*Ca*. Riesia	*Wigglesworthia*	*E. coli*
Cofactors and vitamins	119	203	278	64	130	285
Cell wall and capsule	90	254	185	35	67	261
Virulence, disease, and defense	27	100	100	2	21	98
Potassium metabolism	20	32	24	6	10	45
Photosynthesis	0	0	0	0	0	0
Membrane transport	27	71	138	4	10	127
Iron acquisition and metabolism	0	10	41	0	0	41
RNA metabolism	112	218	190	42	69	246
Nucleosides and nucleotides	40	123	116	14	51	132
Protein metabolism	112	240	213	133	157	242
Cell division and cell cycle	24	41	37	0	24	37
Motility and chemotaxis	42	115	139	0	36	61
Regulation and cell signaling	30	120	130	0	1	171
Secondary metabolism	0	0	4	0	0	26
DNA metabolism	72	179	118	18	30	151
Fatty acids, lipids, and isoprenoids	65	79	106	44	50	103
Nitrogen metabolism	24	24	28	0	0	68
Dormancsy and sporulation	1	3	3	1	1	6
Respiration	59	121	119	12	27	211
Stress response	53	137	146	26	38	240
Metabolism of aromatic compounds	1	7	18	2	0	6
Amino acids and derivatives	184	416	489	39	40	401
Sulfur metabolism	38	43	75	2	2	53
Phosphorus metabolism	38	46	59	5	5	52
Carbohydrates	106	663	614	41	33	759

Supplementary Data: partial sequences of *coaA* generated using aTRAM software and translated sequence of *panD* pseudogene identified manually

>partial_sequence_*coaA*-1
TGAAGTGGCAGAGATTTATCTTCCACTGTCGCGTCTGCTCAACTTTTATATAA
GTTCCAATCTGTGTCGACAGACGGTACTAGAACAGTTTCTCGGTACCGACGGT
CAGAGGATACCTTATGTCATTGGCATCGCCGGTAGCGTGGCAGTTGGCAAGA
GCACCACGGCGCGTGTCCTGCAGGCGCTACTGAGTCGCTGGCCCGAACACCG
TTCGGTGGAGCTAGTGACGACCGACGGGTTTTGCATCCCAACCAGGTACTC
AAACAGCGGGACCTCATGAAGAAGAAGGGGTTCCCGGAGTCTTATGATATCC
ACAGCCTGGTCAATTT

>partial_sequence_*coaA*-2
TAACAGCCCCGGTCTATTCCCATTTGATCTACGACGTCGTCCCCGACGAACAA
AAAATCATTTCCCAACCGGACATTTTGATCTTAGAGGGTTTGAATGTTTTACA
AAGCGCTAGCGATTATGACCAGGATCCGCACCACGTGTTCGTCTCCGACTTTG
TCGACTTTTCCATCTA

>candidate *panD* pseudogene translated sequence
MQRTMLRGKLHRAHVTQADRSPLRRLLRHRSGLSRCGGDLGV*KAIYIYNVDN
GRRFSLYAITDEQRIAHHFGELRCCAPASTIC*LSSPICKCWMKRRFTTPRSSPISTS
TINCSASPKRYYHWCNVT*

1. **Smith WA, Oakeson KF, Johnson KP, Reed DL, Carter T, Smith KL, Koga R, Fukatsu T, Clayton DH, Dale C.** 2013. Phylogenetic analysis of symbionts in feather-feeding lice of the genus *Columbicola*: evidence of repeated symbiont replacements. BMC Evolutionary Biol **13**:109.
2. **Edgar RC.** 2004. MUSCLE: multiple sequence alignment with high accuracy and high throughput. Nucl Acids Res **32**:1792-1797.
3. **Overbeek R, Begley T, Butler RM, Choudhuri JV, Chuang HY, Cohoon M, de Crecy-Lagard V, Diaz N, Disz T, Edwaards R, Fonstein M, Frank ED, Gerdes S, Glass EM, Goesmann A, Hanson A, Iwata-Reuyl D, Jensen R, Jamshidi N, Krause L, Kubal M, Larsen N, Linke B, McHardy AC, Meyer F, Neuweger H, Olsen G, Olsen R, Osterman A, Portnoy V, Pusch GD, Rodionov DA, Ruckert C, Steiner J, Stevens R, Thiele I, Vassieva O, Ye Y, Zagnitko O, Vonstein V.** 2005. The subsystems approach to genome annotation and its use in the project to annotate 1000 genomes. Nucleic Acids Res **33**:5691-5702.
4. **Aziz RK, Bartels D, Best AA, DeJongh M, Disz T, Edwards RA, Formsma K, Gerdes S, Glass EM, Kubal M, Meyer F, Olsen GJ, Olson R, Osterman AL, Overbeek RA, McNeil LK, Paarmann D, Paczian T, Parrello B, Pusch C, Reich C, Stevens R, Vassieva O, Vonstein V, Wilke A, Zagnitko O.** 2008. The RAST server: rapid annotations using subsystems technology. BMC Genomics **9**:75.
